# From wellness to medicine: Geroscience, biomarkers and the translational emergence of longevity medicine

**DOI:** 10.1016/j.fhj.2026.100564

**Published:** 2026-09-08

**Authors:** Chloé A. Fasel, Louis J. Koizia, Benjamin H.L. Harris

**Affiliations:** a School of Neuroscience, King's College London, London, UK; b Cutrale Perioperative and Ageing Group, Department of Bioengineering, Imperial College London, London, UK; c St. Catherine’s College, University of Oxford, Oxford, UK; d Department of Oncology, University of Oxford, Oxford, UK; e Institute for Liver and Digestive Health, Division of Medicine, University College London, London, UK; f Department of Psychosocial Rehabilitation, Medical University of Lodz, Lodz, Poland; g Collaborating Centre for Values-based Practice, St Catherine's College, Oxford, UK

**Keywords:** Longevity medicine, Biomarkers, Geroscience, Healthspan, Preventive medicine, Healthy Ageing, Inflammaging, Wellness

## Abstract

Longevity medicine has emerged at the intersection of geroscience, biomarker science and preventive healthcare, yet its origins and relationship to conventional medicine remain incompletely understood. This article examines how advances in ageing biology transformed ageing from an inevitable consequence of life into a measurable and potentially modifiable determinant of health. Historically, medicine has focused on the diagnosis and treatment of established disease, limiting its ability to address gradual biological decline before pathology develops. Geroscience challenged this paradigm by identifying shared biological mechanisms that contribute to ageing and multiple chronic diseases, while advances in biomarker science enabled ageing-related processes to be quantified in living humans. However, the ability to measure ageing biology developed more rapidly than the clinical frameworks required to act upon it. We argue that the emergence of longevity-focused services outside traditional healthcare systems represents a pre-institutional translational phase in the evolution of longevity medicine. The integration of ageing biology into clinical practice will require new clinical, regulatory and educational frameworks to support healthspan-focused preventive care.

## Introduction

Modern medicine excels at the treatment of established disease but remains comparatively limited in its capacity to intervene before pathology develops. Clinical care is largely organised around diagnostic thresholds that determine when biological variation becomes illness, when intervention is justified, and when professional responsibility is formally assumed, resulting in a predominantly reactive rather than proactive model of care. This largely binary, disease-centred approach has delivered substantial gains in survival and acute disease management, yet has been less effective at preserving function and physiological resilience across the decades preceding diagnosis.^1^, ^2^, ^3^

As populations age and the burden of chronic disease increases, many individuals experience progressive declines in metabolic health, cognitive performance, physical capacity or stress tolerance despite remaining within conventional laboratory reference ranges. These gradual, multisystem changes rarely trigger categorical diagnosis, yet cumulatively influence long-term morbidity, disability, and quality of life.^4^, ^5^, ^6^

Advances in geroscience and biomarker science now allow aspects of ageing biology to be measured directly in living humans, transforming age-related decline from an abstract risk factor into a quantifiable biological trajectory.^7^, ^8^, ^9^, ^10^ However, measurement capability has advanced more rapidly than the clinical, regulatory and reimbursement frameworks required to act on such information, limiting its incorporation into routine care.^11^ This widening gap between biological measurement and clinical action provides the context for the emergence of longevity medicine.

For the purposes of this article, we use the term longevity medicine to describe the emerging clinical translation of geroscience and related advances in risk assessment, biomarker science and longitudinal health monitoring, with the aim of preserving function, physiological resilience and preventing disease. This is distinct from geroscience, which primarily seeks to understand the biological mechanisms linking ageing to disease,^7^, ^12^ and from the broader consumer wellness industry, which encompasses a much wider range of health-related products and services with varying levels of supporting evidence. The objective of longevity medicine is therefore not the extension of lifespan for its own sake, but the evidence-based preservation of health and function across a longer period of life. This gap between biological insight and institutional readiness contributed to the early emergence of longevity-focused interventions outside traditional healthcare systems, which this article examines in the context of the historical sidelining of ageing in Western medicine, its reframing through geroscience, and the implications of consumer longevity for clinical reintegration in preventive care.

## The historical marginalisation of ageing in medicine

Modern clinical medicine developed around the identification and management of discrete pathological states. Diseases were defined by observable symptoms, structural abnormalities or deviations from physiological norms that could be reliably diagnosed, treated and monitored within finite timeframes. This framework proved highly effective for acute illness and later for chronic disease management but was not designed to address gradual, system-wide biological change unfolding over decades.^13^

Ageing did not conform to this model. It lacked a singular cause, a clear point of onset and a definitive clinical endpoint. Instead, it manifested as a diffuse accumulation of molecular and cellular alterations affecting multiple organ systems simultaneously. In the absence of a unifying diagnostic criterion, ageing was positioned as a background condition rather than a legitimate clinical target. Moreover, ageing was long regarded as an inevitable and non-modifiable process, further diminishing its perceived relevance for clinical intervention. Ethical concerns also played a role, as efforts to medicalise ageing risked being perceived as ageist or as pathologising a universal life-course process.

Clinical actionability has historically depended on the establishment of thresholds that distinguish normal variation from pathology. Risk states such as hypertension became actionable once population-level cut-offs were defined and linked to downstream disease risk. Age-related biological change, by contrast, progressed along continuous trajectories without agreed inflection points, limiting its incorporation into diagnostic coding, reimbursement structures and clinical guidelines (Fig.1).^14^

**Fig. 1:**
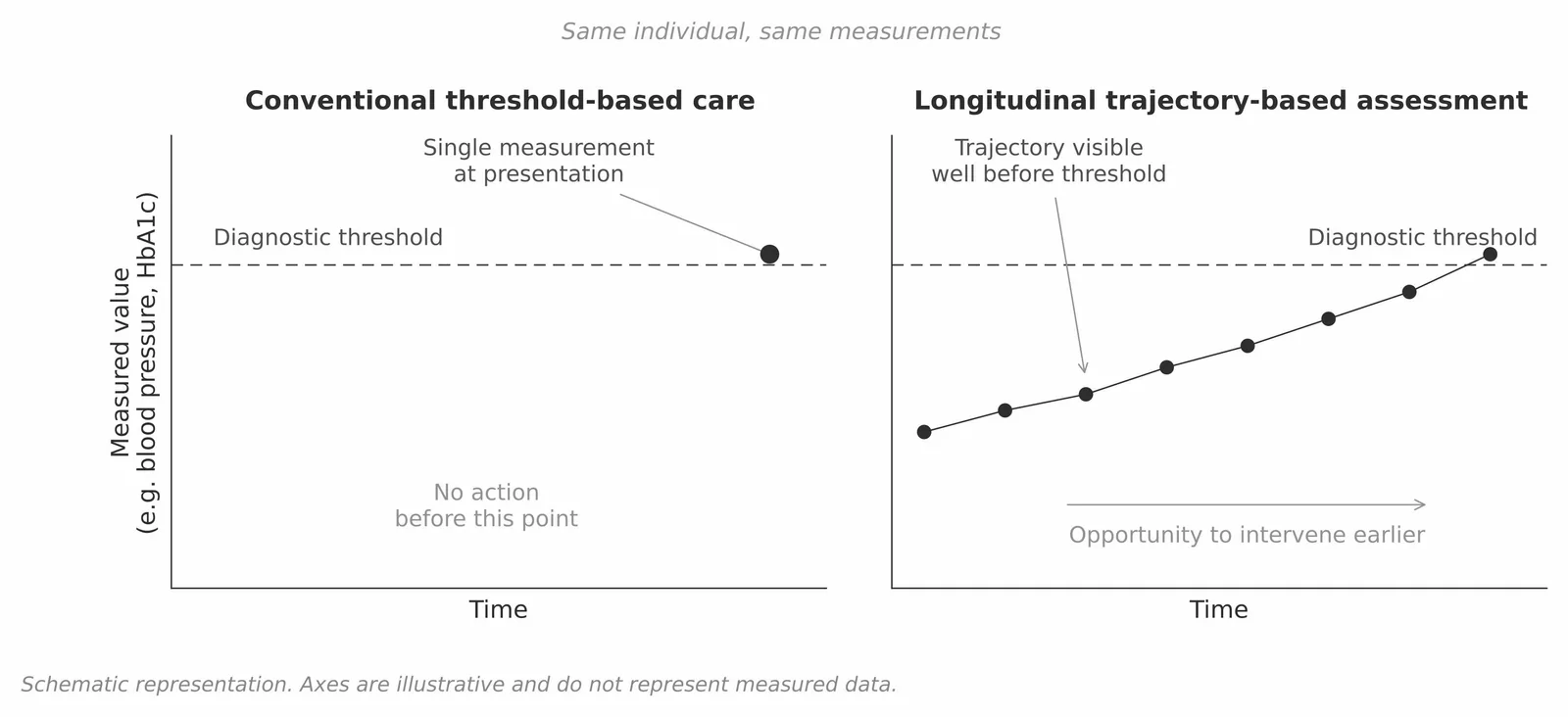
Conventional threshold-based care versus longitudinal trajectory-based assessment. Longitudinal measurements may reveal clinically meaningful change before diagnostic thresholds are crossed, creating opportunities for earlier intervention.

Evidentiary standards also contributed to the limited clinical incorporation of ageing-related interventions. Randomised controlled trials are optimised for interventions with discrete outcomes over limited durations, whereas interventions targeting ageing processes typically require extended time horizons and complex, multifactorial endpoints such as delayed multimorbidity or preserved function. Consequently, while ageing biology was mechanistically compelling, related interventions remained largely outside routine clinical care. This position began to change as advances in ageing biology challenged the assumption that age-related decline was inevitable and non-modifiable, driving growing scientific interest in geroscience.

## The emergence of geroscience and the reframing of ageing as modifiable biology

The desire to extend human life is among the oldest aspirations in medicine, progressing over centuries from the symbolic elixirs and restorative regimens of pre-modern traditions toward an empirical science of ageing.^15^ Over the past several decades, geroscience has emerged as an interdisciplinary field integrating the biology of ageing with chronic disease research, challenging the traditional view of ageing as an inevitable and untreatable process. Important foundations for the scientific study of ageing were laid by Metchnikoff and Korenchevsky^16^, ^17^ in the early twentieth century, while renewed momentum emerged in the early 2000s through institutional initiatives such as the trans-NIH GeroScience Interest Group led by Felipe Sierra. This culminated in the publication of *The Emergence of Geroscience*, which articulated the geroscience hypothesis that ageing is a major driver of multiple chronic diseases and therefore represents a strategic therapeutic target for extending healthspan.^12^

Through this lens, geroscience reframed ageing as a biologically regulated phenomenon that shapes vulnerability to disease across multiple organ systems, rather than as an incidental correlate of pathology. Ageing biology was positioned as an upstream determinant of health outcomes.^12^

Multiple chronic diseases were shown to share common upstream mechanisms, including chronic inflammation, mitochondrial dysfunction, impaired proteostasis, genomic instability, altered nutrient sensing and cellular senescence. These processes accumulate with age and precede overt disease onset, contributing to progressive functional decline across tissues.^18^, ^19^, ^20^, ^21^ Furthermore, disease development was increasingly understood to reflect cumulative physiological changes shaped not only by intrinsic biology but also by environmental, behavioural and social exposures across the life course, rather than solely isolated singular events.^22^ This systems-level perspective challenged traditional episodic models of disease and supported a more holistic, integrative approach to health and care. It also carries an evolutionary dimension, the human lifespan having lengthened substantially as successive populations gained protection from the infectious, inflammatory and nutritional pressures that historically shaped the diseases of later life.^23^

Experimental evidence further demonstrated that modulation of ageing-related pathways could delay disease onset and preserve function across multiple systems in animal models. These findings established biological plausibility for intervening on ageing processes prior to disease manifestation and strengthened translational relevance as analogous mechanisms became measurable in humans.^24^

Importantly, geroscience does not require the reclassification of ageing as a disease. Instead, it conceptualises ageing-related decline as a modifiable state of elevated biological risk. Medicine already intervenes in non-disease states when causal links to downstream pathology are established, such as impaired glucose tolerance. Geroscience extends this preventive logic across disease boundaries.

## The biological quantification of ageing

The translational potential of geroscience expanded substantially with advances in biomarker science, which rendered ageing biology observable and trackable in living humans. These developments provided increasingly practical means of characterising and tracking biological change associated with ageing in living humans.^8^

Ageing-related biomarkers may differ in their intended application from many traditional diagnostic markers. Rather than relying on cross-sectional thresholds to define pathology, they are most informative when assessed longitudinally within individuals, with change over time serving as the primary indicator of biological progression or resilience.^9^

Composite biological age measures, inflammatory profiles, metabolic indices and physiological resilience metrics provide systems-level insight into individual ageing trajectories. Such tools may identify biologically meaningful change before overt disease diagnosis, highlighting the limitations of disease-centred, threshold-based clinical assessment.^10^

Among emerging biological age measures, epigenetic clocks based on DNA methylation patterns and glycan-based biomarkers reflecting immune and inflammatory ageing have gained particular prominence. Methylation-based clocks^9^, ^25^ provide sensitive measures of age-related molecular change and may reflect the cumulative influence of biological and environmental exposures across the life course, while glycan profiles^26^ provide dynamic markers of age-related changes in immune and inflammatory function. Together, these complementary approaches illustrate the heterogeneity of ageing biology and the potential value of multi-dimensional assessment.

The extent of validation varies substantially across these measures, reflecting differences in how validation is defined and assessed, as well as the context in which validation is undertaken. In summary, first-generation epigenetic clocks, including the Horvath clock, were developed primarily to estimate chronological age from DNA methylation patterns,^10^ while subsequent measures such as PhenoAge and GrimAge incorporated clinical or mortality-related information to improve prediction of ageing-related outcomes.^9^, ^27^ Measures of the pace of ageing, such as DunedinPACE, take a related but distinct approach by attempting to quantify the rate of biological change over time.^28^ Glycan-based measures, particularly patterns of immunoglobulin G glycosylation, have similarly demonstrated associations with chronological age, inflammation and age-related phenotypes.^26^ Telomere length, most commonly measured in leucocytes, has been studied for longer than any of these measures, but high measurement variability and inconsistent prediction have been described.^29^ Conventional inflammatory, metabolic and physiological measures may also provide complementary information regarding ageing-related risk. However, validation as a correlate or predictor of ageing-related outcomes is distinct from validation as a clinical decision-making tool.

Although several of these measures have demonstrated associations with morbidity, mortality and functional outcomes across independent populations, evidence that their routine measurement provides clinically meaningful information beyond established risk factors, identifies an effective intervention or improves patient outcomes remains limited. No single biological-age biomarker currently provides a universally accepted clinical measure of ageing. Mass clinical translation will therefore require prospective validation of reproducibility, incremental predictive value, responsiveness to intervention and, importantly, evidence that biomarker-guided management improves meaningful health outcomes. A summary of the principal approaches and their current evidence base is provided in Table 1.

**Table 1:** Approaches to the biological quantification of ageing.

Biomarker approach	Example	What it measures	Evidence and validation	Exemplar references
DNA methylation: chronological clocks	Horvath clock	Age-associated methylation patterns	Validated against chronological age	10, 25
DNA methylation: phenotypic clocks	PhenoAge, GrimAge	Mortality and health-related methylation signatures	Associated with morbidity and mortality in multiple cohorts	9, 27
DNA methylation: pace of ageing	DunedinPACE	Rate of biological ageing over time	Validated against ageing-related phenotypes and outcomes	^28^
Telomere length	Leucocyte telomere length	Replicative ageing and cumulative cellular stress	Associated with age and mortality, but high measurement variability and weak prediction in individuals	^29^
Glycomic	IgG glycosylation	Immune and inflammatory ageing	Associated with age and inflammatory phenotypes	^26^
Conventional and composite	Inflammatory, metabolic and physiological measures	Multisystem physiological risk	Individual components (CRP, IL-6, HbA1c, eGFR, blood pressure, gait speed) well validated; composite indices far less so	^11^

Importantly, the kinetics of these biomarkers differ, with some measures demonstrating responsiveness over months following targeted interventions, while others reflect longer-term biological change. This has prompted growing interest in optimising testing intervals to balance biological signal with clinical practicality, particularly in the context of lifestyle, pharmacological or behavioural interventions aimed at modifying ageing trajectories.

However, the availability of longitudinal biological information outpaced institutional readiness to act upon it. In the absence of consensus regarding intervention thresholds, clinical responsibility, reimbursement structures or optimal monitoring frameworks, biologically meaningful data increasingly migrated to settings outside traditional healthcare systems.

## Consumer longevity as a translational response

Consumer-facing longevity systems have emerged, in part, in response to the widening gap between the growing availability of biomarker-based health information and the capacity of conventional healthcare systems to interpret and act upon it. As such assessments have become increasingly accessible, individuals can obtain information about biological ageing and future health risk for which established clinical pathways, thresholds and interventions may not yet exist.

This gap has provided fertile ground for consumer-facing longevity services, reinforced by a broader shift towards the marketisation and individualisation of health and wellbeing. Over recent decades, responsibility for maintaining health has increasingly extended beyond traditional healthcare institutions towards individuals taking an active interest in their health. There are growing private markets for wellness, prevention, self-tracking and personalised health technologies. Indeed, Union Bank of Switzerland (UBS) estimates that the total annual market opportunity linked to longevity, spanning healthcare, consumer, financial services and real estate, will grow to $8tn by 2030.^30^ This environment has provided considerable capital and consumer demand for rapid innovation, enabling and driving emerging biomarkers and monitoring technologies to move from research settings into commercial use before equivalent pathways have developed within conventional healthcare. Such market mechanisms can accelerate translation and experimentation, but commercial availability should not be conflated with demonstrated clinical utility.

Direct-to-consumer biomarker and multi-omic testing has brought blood-based panels, epigenetic and metabolomic profiling and inflammatory and glycomic markers within individual reach. Whilst consumer wearables and continuous monitoring have extended measurement of cardiovascular, metabolic, and sleep physiology into everyday life. Longevity-oriented clinics and membership services increasingly integrate these technologies with periodic screening, advanced imaging and structured clinical interpretation, frequently coupled with lifestyle, nutritional and pharmacological interventions intended to modify health trajectories rather than simply treat established disease.

A typical consumer longevity pathway illustrates how this model differs from conventional episodic care. An individual without established disease may undergo a baseline assessment incorporating clinical history, lifestyle and functional measures, conventional cardiovascular and metabolic risk factors, genomic profiling, advanced imaging and emerging biomarkers of biological ageing, with wearable-derived data integrated longitudinally to monitor changes in health and function over time. These findings may then be integrated into an individual risk profile and used to inform lifestyle or other preventive interventions, followed by repeat assessment over subsequent months or years. Rather than asking whether a diagnostic threshold has been crossed at a single time point, this model places greater emphasis on whether an individual’s biological trajectory is changing over time.

For example, an individual may remain below conventional thresholds for hypertension or diabetes while serial measurements demonstrate progressively increasing blood pressure, worsening glycaemic control, declining physical fitness and changes in ageing-related biomarkers. Considered longitudinally, these changes could indicate an adverse trajectory before a categorical diagnosis is reached, and provide an opportunity to reinforce established preventive measures. However, while the conventional risk factors within such a profile have established clinical significance, the additional value of many emerging biological-age measures in determining treatment or improving individual outcomes remains uncertain.

The emergence of dedicated brain health clinics, the growth of commercial whole-body screening services such as Neko Health,^31^ and the increasing use of whole-body imaging within private preventive health systems^32^ provide illustrative examples of this trend. These offer an intuitively attractive proposition: identifying pathological or adverse biological changes before symptoms or conventional diagnostic thresholds are reached. However, improved detection of abnormalities does not necessarily translate into improved outcomes. Advanced imaging may identify clinically important disease, but can also generate incidental findings and overdiagnosis, which may in turn drive anxiety, further investigation and downstream healthcare expenditure. In individuals with otherwise unexplained symptoms, incidental abnormalities may also be incorrectly attributed as causal, potentially reinforcing illness beliefs and behaviours and prompting further investigation and healthcare utilisation. Nevertheless, incidental findings should not be regarded as uniformly devoid of clinical value. In a meta-analysis of 32 studies involving apparently asymptomatic adults undergoing brain and body magnetic resonance imaging, the pooled prevalence of potentially serious incidental findings was 3.9% (95% CI: 0.4%–27.1%), although only around one fifth of potentially serious incidental findings ultimately resulted in a serious final diagnosis.^33^ This creates an increasingly important tension between population-level and individual benefit: an approach that cannot be justified as population-wide screening may nevertheless identify clinically important disease in a particular individual. Longevity medicine is increasingly occupying this space (Fig. 2), where preferences for earlier and more intensive risk detection may extend beyond what can currently be justified within population-based healthcare.

**Fig. 2:**
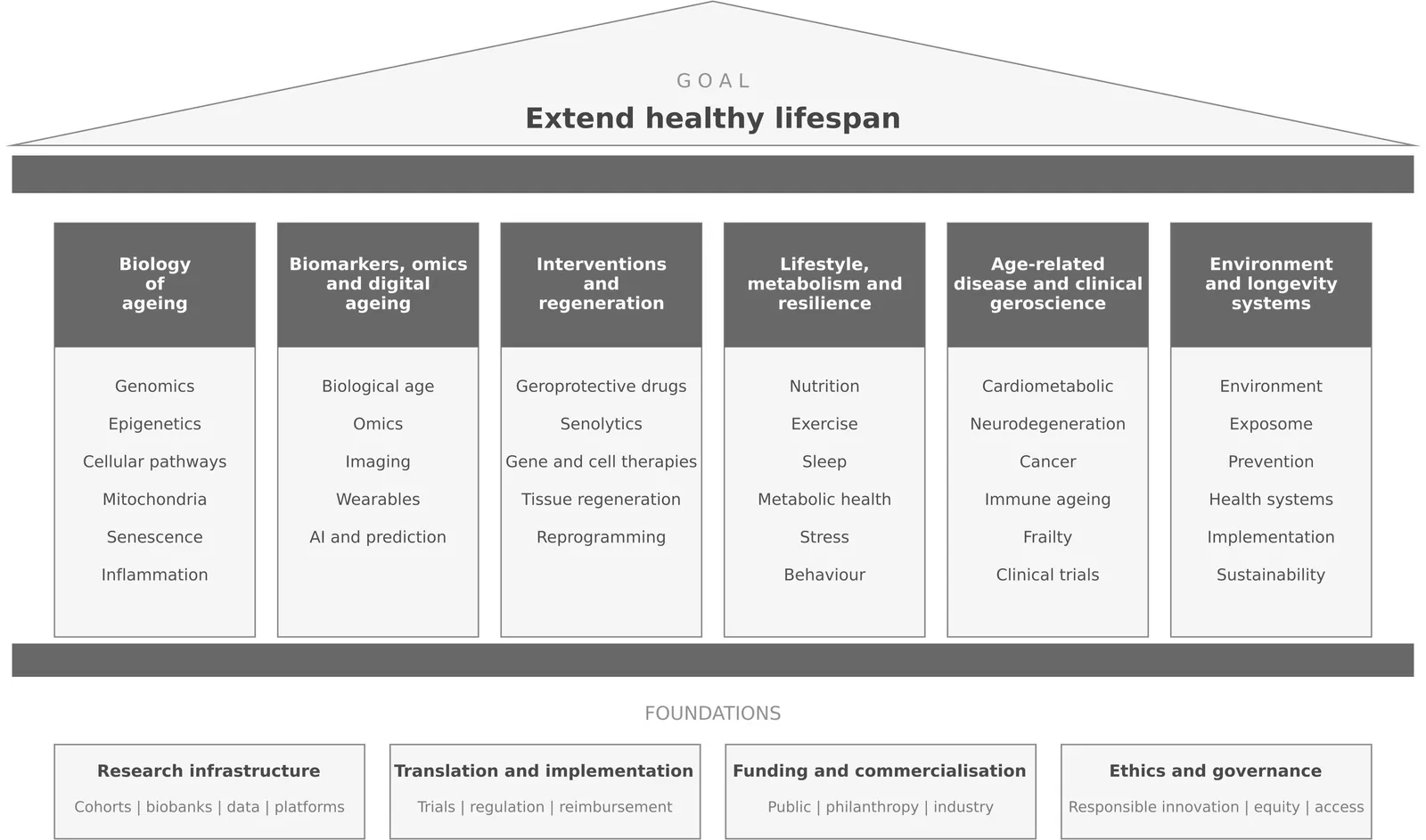
Six scientific pillars support the goal of extending healthy lifespan, underpinned by cross-cutting foundations in infrastructure, translation, funding, and governance.

The implications of this individualised model may also extend beyond those who directly purchase these services. Abnormal or incidental findings generated through consumer-initiated testing may lead individuals back into conventional healthcare for interpretation, confirmatory testing, surveillance or specialist referral. Privately purchased screening can therefore generate downstream costs for publicly funded systems, particularly where the clinical significance of detected abnormalities is uncertain.^34^, ^35^ Evaluation of longevity-focused approaches should therefore account for downstream healthcare utilisation rather than direct cost alone.

Despite these limitations, by assembling repeated measurements into individual longitudinal records, longevity services can create practical and frequent feedback loops between assessment and action that conventional episodic care can rarely provide. They function, in effect, as real-world environments in which approaches to interpreting and acting upon ageing-related biology are being tested at scale, albeit unevenly and largely within organisational and commercial silos. At present, uptake appears to be concentrated among health-engaged and frequently higher-income populations, although robust evidence characterising the demographic and socioeconomic reach of longevity-specific services remains limited. Cost, digital access, health literacy, the time required for repeated monitoring and the capacity to act on recommendations may all influence who participates and who ultimately derives benefit. This raises an important question of whether emerging approaches to biological ageing can translate beyond relatively advantaged early adopters to populations experiencing the greatest burden of age-related disease. Without deliberate attention to accessibility and external validity, such approaches could widen rather than reduce existing inequalities in healthy ageing. Demonstrating their acceptability, validity and clinical utility across socioeconomically, ethnically and geographically diverse populations will therefore be important for wider implementation.

Consumer longevity may therefore function as a pre-institutional translational layer rather than an alternative to medicine. While heterogeneous in quality and oversight, these systems demonstrate both the feasibility of acting on ageing biology and the risks of doing so without consistent clinical governance.

This unevenness is both the strength of the consumer model and its principal limitation. Provision ranges from analytically robust testing accompanied by careful interpretation to offerings whose analytical and clinical validity is poorly established, while the interventions attached to them vary correspondingly in evidence and oversight. Commercial incentives, the potential for overinterpretation of borderline results, and the management of incidental findings further complicate matters, underscoring why durable translation will ultimately depend on integration with established clinical standards rather than consumer provision alone.

## Clinical re-integration and implications for preventive care

Elements of longevity science are now re-entering formal medical practice through preventive and specialist domains. Preventive cardiology, metabolic medicine, frailty assessment and cognitive health are increasingly representing early points of clinical engagement.^32^, ^36^ Clinicians are increasingly required to interpret longitudinal biological data and engage with pre-disease risk states, raising questions of evidence, professional responsibility, and scope of practice. Integration remains uneven, driven by regulatory uncertainty, healthcare system constraints and the absence of established clinical pathways for acting on ageing-related biological risk.

The integration of ageing-related biomarkers also raises broader ethical and health-system considerations. Increased testing may medicalise aspects of normal ageing and generate findings of uncertain significance. In healthcare systems operating with finite resources, investment in biological-age assessment inevitably carries opportunity costs.

A further distinction is required between measuring ageing trajectories and demonstrating that they can be meaningfully modified. At a population level, some of the most established opportunities to promote healthier ageing remain interventions targeting well-characterised determinants of chronic disease, including smoking, physical inactivity, poor diet, obesity, hypertension and metabolic risk. By contrast, evidence that interventions selected or modified on the basis of current biological-age measurements provide additional clinical benefit at scale remains limited. Moreover, measurement alone does not necessarily prompt behavioural or clinical action and, even where it does, such action may not translate into improved outcomes.

The potential value of individualised biological measurement should also be distinguished from its capacity to improve health at the population level. Even where individual preventive interventions are evidence-based, their population impact may be constrained by limited reach and engagement. In PRODEMOS, for example, a coach-supported mobile intervention targeting established dementia risk factors produced only modest improvement in risk score among participants, and recruitment of the underserved populations it was designed to reach proved challenging.^37^ More fundamentally, targeting those identified as being at highest risk will inevitably miss disease arising across the much larger population at lower individual risk. In the EPIC-Norfolk cohort, approximately seven in ten incident dementia cases arose among individuals who would not have been classified as high risk at baseline.^38^ These observations reflect the broader prevention principle that individually targeted approaches, even when effective for those who receive them, may produce only modest shifts in disease incidence across whole populations. Individualised assessment of ageing biology should therefore be viewed as complementary to, rather than a substitute for, population-level interventions addressing the social, environmental and behavioural determinants of healthy ageing.

Regulatory challenges arise at several levels. Ageing-related tests may be offered as consumer wellness products while generating information that individuals and clinicians subsequently use to make health decisions, creating uncertainty around the boundary between consumer information and medical practice. Standards for analytical validity, clinical validity and, crucially, clinical utility also vary across emerging biomarkers, while responsibility for interpreting results generated outside conventional healthcare may be unclear. Longitudinal biological data additionally raise questions regarding privacy, data ownership and secondary use, particularly where information is collected repeatedly through commercial platforms.

Addressing these challenges will require regulatory frameworks proportionate to the clinical consequences of the information generated. This could include clearer evidentiary standards for biomarkers used to inform clinical decisions, transparent communication of uncertainty and limitations, appropriate oversight of health claims, robust data-governance requirements, and defined pathways for managing findings of uncertain significance. Importantly, regulation should distinguish between demonstrating that a biomarker can be measured accurately, that it predicts an outcome, and that using it to guide care produces meaningful clinical benefit. Routine implementation, particularly within resource-constrained healthcare systems, will require prospective evidence that biomarker-guided care provides additional benefit over existing preventive pathways, alongside formal health-economic evaluation.

Educational gaps further compound these challenges. Undergraduate and postgraduate medical curricula have historically placed greater emphasis on disease diagnosis and management than on the biology of ageing, systems-level risk, or longitudinal biomarker interpretation, and teaching in ageing and geriatric medicine remains variable and, in many countries, non-mandatory.^39^ As a result, many clinicians lack formal training in geroscience principles and the practical application of ageing-related biomarkers, limiting confidence and consistency in clinical implementation.

Extending clinical responsibility upstream will therefore require not only adaptation of preventive frameworks and clearer standards for action on longitudinal risk, but also targeted educational reform to equip current and future clinicians with the competencies needed to integrate ageing biology into routine care.

## Conclusion

Longevity medicine emerged through consumer translation not because it lacked biological legitimacy, but because healthcare systems were not yet positioned to act on pre-disease biological information. Historical disease-centred frameworks, evidentiary structures, and regulatory models constrained clinical engagement with gradual, system-wide biological change, even as geroscience reframed ageing as a modifiable driver of chronic disease and biomarker science enabled its quantification in living humans.

Understanding consumer longevity as a pre-institutional translational phase provides a coherent framework for interpreting its rapid growth and heterogeneous practices. While these systems demonstrated the feasibility of acting on ageing biology, their consumer-facing implementation also generated scepticism regarding scientific rigour, clinical credibility, and commercial influence, highlighting the delicate balance between innovation and evidence-based care.

As elements of longevity science are re-integrated into formal medical practice, the challenge for healthcare systems is to extend preventive care upstream in a responsible and equitable manner. This will require alignment between biological insight and institutional mandate, the development of clear clinical pathways for acting on longitudinal risk, strengthened governance structures, and educational reform to equip clinicians with the competencies necessary to interpret and apply ageing-related biomarkers. Equally, it will require evidence that measurement changes management and improves patient-centred outcomes, together with honest appraisal of the potential harms, inequities and opportunity costs that accompany extending assessment into asymptomatic populations.

Ultimately, the integration of geroscience into preventive medicine offers an opportunity to shift healthcare from a predominantly reactive model toward one focused on preserving function, resilience, and healthspan across the life course. Achieving this transition will depend not only on scientific advances, but also on the evolution of clinical, regulatory, and educational frameworks capable of translating ageing biology into safe and effective care.

## CRediT authorship contribution statement

**Chloé A. Fasel:** Writing – review & editing, Writing – original draft, Conceptualization. **Louis J. Koizia:** Writing – review & editing, Writing – original draft, Conceptualization. **Benjamin H.L. Harris:** Writing – review & editing, Writing – original draft, Conceptualization.

## Ethics approval and consent to participate

Not applicable.

## Funding

BHLH would like to thank the Orthogeriatric Research Fund for their support.

## Declaration of Competing Interest

The authors declare that they have no known competing financial interests or personal relationships that could have appeared to influence the work reported in this paper.
